# Amino-POSS Grafted Polyimide-Based Self-Stratifying Composite Coatings for Simultaneously Improved Mechanical and Tribological Properties

**DOI:** 10.3390/polym18010045

**Published:** 2025-12-24

**Authors:** Chuanyong Yu, Peng Zhang, Min Wei, Qiwei Wang, Wei Zhang

**Affiliations:** 1 School of Mechatronic Engineering and Automation, Foshan University, Foshan 528225, China; yucy14@163.com (C.Y.); zhangwei18@fosu.edu.cn (W.Z.); 2 Institute of Advanced Wear & Corrosion Resistant and Functional Materials, Jinan University, Guangzhou 510632, China; tzhangpeng@jnu.edu.cn

**Keywords:** polyimide-based composites, self-stratifying gradient coatings, organic-inorganic hybrid, mechanical properties, friction and wear

## Abstract

The development of emerging high-tech technologies comes with a growing demand for composite materials with outstanding mechanical properties and wear resistance. Herein, we fabricated organic-inorganic self-stratifying gradient coatings based on silicon density by chemically bonding octa- and mono-amino polyhedral oligomeric silsesquioxane (POSS) onto the polyimide (PI) resin. The microstructure and chemical characteristics of POSS-PI-based composite coatings were investigated. The enhancements to the mechanical properties and wear resistance of the PI-based composites due to the gradient structure were also investigated. As expected, the addition of POSS significantly increased the composites’ thermal stability and mechanical properties. In particular, the tensile strength and nano-indentation hardness of the 4 wt.% POSS-PI composites were enhanced by 28.6% and 68.4%, respectively. Furthermore, compared with that of pure PI, the wear rate of the POSS-PI self-stratifying coatings decreased by 78.9%, which was due to the enhanced cross-linking density and gradient structure that resulted from the self-stratifying of POSS.

## 1. Introduction

Due to their excellent self-lubrication performance, high strength, and high stiffness, polymer-based composite coatings have been used to effectively control friction and wear in mechanical equipment [1,2,3]. Polyimide (PI) is one of the polymers most widely used in such coatings, particularly for spacecraft, automotive, and aircraft industries, owing to its high thermal stability, outstanding mechanical properties, and chemical inertness [4,5]. However, traditional PI-based composites have proven to be insufficient for the long-term protection of metal substrates from friction and wear under increasingly harsh service conditions, and their comprehensive properties need to be further improved [6,7].

From recent research reports, scholars have mainly reinforced the coatings by incorporating inorganic nanoparticles or 2D thin-layered nano-materials [8,9]. Various inorganic nanoparticles such as aluminum oxide nanoparticles (Al_2_O_3_) [10], graphitic carbon nitride (g-C_3_N_4_) [11], and graphene nano-sheets [12] are considered to be promising in this polymeric field. Silica-based nanoparticles are also especially promising since they can be added into the PI matrix to form a functional silica/PI composite film, which could improve the thermal stability and mechanical properties [13,14]. However, achieving good dispersion and high interfacial bonding strength between the nano-fillers and the PI matrix is a major challenge in the development for these applications, as well as the weak load-bearing capacity and poor wear resistance [15,16,17]. Because of these critical issues, the development and application of PI-based composites are severely restricted in friction and wear under harsh conditions.

Recently, self-stratifying gradient composite coatings, as novel and modern composite materials, have drawn wide attention from scientific researchers all over the world. By utilizing the phase separation of immiscible polymers, researchers have fabricated multi-functional self-stratifying coatings with a gradient structure via a single coating process. During the drying and curing processes, one resin component migrates to the free surface, while the other bonds with the substrate. This self-stratifying gradient structure could improve the hardness and wear resistance and reduce the interfacial problem of composite coatings [18,19]. Notably, polyhedral oligomeric silsesquioxane (POSS) possesses an inorganic “cage-like” framework consisting of Si-O-Si bonds surrounded by various organic functional groups, such as inert groups (phenyl and alkyl groups) and active groups (amino, hydroxyl, and vinyl groups) [20,21,22]. Furthermore, POSS can be chemically added to polymeric materials and achieve different microstructures according to its number of functional groups: POSS with multi-functional groups can be chemically bonded onto the polymer matrix, acting as a cross-linking agent that is mainly distributed inside of the polyimide system, while mono-functional POSS can be added as an end-capping agent that is mainly distributed on the composite surface [23,24]. This strategy of chemical bonding solves the dispersion and agglomeration problems with the nanoparticles in the polymers and fabricates a self-stratifying coating with a continuous gradient in its structure and properties. Moreover, the modified polymer can provide the excellent processability and toughness of polymer materials while also retaining the outstanding heat resistance, corrosion resistance, and excellent mechanical properties of inorganic materials [25]. Yuan et al. [26] reported that the amino-POSS was grafted onto Nomex/PTFE fabric significantly improved interfacial adhesion and UV resistance. Yari et al. [27] reported that the octa-hydroxyl POSS can improve the cross-linking density and hardness of the acrylic resin-based composite coatings. Therefore, the multi-functional POSS can be utilized to fabricate the composite coatings with unique microstructure.

Bearing the above in mind, in this study, both octa- and mono-amino POSS were covalently grafted onto the PI matrix to fabricate self-stratifying gradient composite coatings. Various experimental methods were used to determine the morphology and structural characteristics of the POSS-PI-based gradient coatings. Additionally, the effect of microstructures on the thermal stability, mechanical properties, and tribological performance of the PI-based gradient coating was further studied. The results indicated that the POSS-PI gradient structure greatly enhanced the thermal stability, mechanical properties, and wear resistance of the PI-based materials, which provides new research ideas for the realization of multi-functional coatings.

## 2. Materials and Methods

### 2.1. Materials

Both octa-amino-phenyl POSS (OPOSS) and mono-amino hepta-isobutyl POSS (MPOSS) were provided by Huawei Ruike Co., Ltd. (Agency of Hybrid Plastic Corporation, Hattiesburg, MS, USA). Shanghai Kefeng Chemical Reagent Co., Ltd. (Shanghai, China) provided us with the 4, 4-Diaminodiphenyl ether (ODA). Pyromellitic dianhydride (PMDA) was supplied by Sinopharm Group Chemical Reagent Co., Ltd. (Shanghai, China). N, N′-dimethylacetamide (DMA), and ethyl alcohol were supplied by Tianjin Chemical Reagent (Tianjin, China). All the chemical reagents used in this study were of analytical grade.

### 2.2. Fabrication of POSS-PI-Based Gradient Coatings

**Figure 1:**
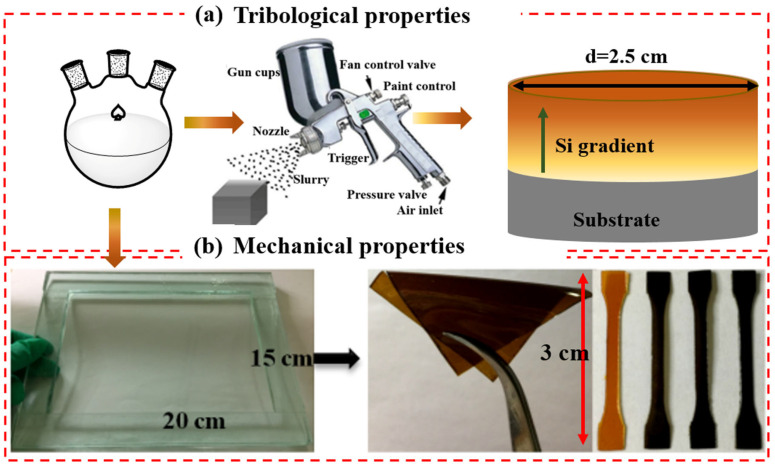
Preparation routes for the POSS-PI composite films and coatings.

The PI-precursor solution (PAA) was synthesized from PMDA and ODA, as described in our previous work [2]. Then, both the octa- and mono-amino POSS (OMPOSS) were introduced into the PAA mixture and stirred for 40 min. The weight compositions of the OMPOSS-PAA mixtures are listed in Table 1. The POSS-PAA mixture was sprayed onto a steel block substrate (316 L) that had been sandblasted and cleaned in advance, then cured at 135 °C for 2 h and 280 °C for 2 h. The gradient composite coatings were prepared successfully via the routes shown in Figure 1. The average thickness of the OMPOSS-PI gradient composite coatings was 25 ± 3 μm.

### 2.3. Characterization of the POSS-PI Composite Coatings

The transmission electron microscopy (TEM, FEI TECNAL TF 20, 200 kV) (FEI Co., Ltd., Portland, OR, USA) was used to study the microstructure of POSS and POSS-PI composites. The Nexus 870 Fourier Infrared Spectrometer was used to chemically analyze the functional groups of functionalized POSS-PI, with a scanning range from 400 cm^−1^ to 2000 cm^−1^. The X-ray diffraction (XRD) patterns of the POSS-PI hybrid composites were recorded within a 2θ range of 5–60° using a Philips XRD device (PANalytical B.V., Almelo, The Netherlands, Cu radiation). X-ray photoelectron spectroscopy (XPS, ESCALAB 250Xi electron diffraction spectrum) (Thermo Fisher Scientific, Portland, OR, USA) and XPS etch tests were used to determine the chemical state of elements in the composites under the conditions of ion energy of 2000 eV, a sputter rate (Ta_2_O_5_:) of 0.18 nm/s, and etch cycle of 120 s for 5 times. For thermogravimetric analysis (TGA) tests, an STA449C thermogravimetric analyzer (Netzsch Grinding and Dispersing, Bayern, Germany) was used under a N_2_ atmosphere with a heating rate of about 10 °C/min; and the test temperature range was from room temperature to 800 °C.

### 2.4. Mechanical Tests and Tribological Properties

The static water contact angles were determined by a contact angle goniometer (OCA15, Dataphysics Company, Stuttgart, Germany). The ultrapure water (twice distilled) with a volume of about 5 μL was dropped onto the coatings surface. The tensile strength performance of the POSS-PI-based composites was determined by using a model WDW-200 universal testing machine on 3 × 0.015 mm^3^ dumbbell-shaped specimens. The tearing specimens were right-angled, and the tearing tests were repeated 3 times for each group of specimens. The dynamic thermal analysis (DMA) curves of the sample were measured on a dynamic thermal analyzer (Netzsch, 242E, Bayern, Germany) at a heating rate of 5 °C/min, for a test temperature range from 25 to 450 °C, and at a frequency of 1 Hz. The morphologies and cross-sectional profiles of the coating wear tracks were analyzed using a non-contact surface profiler (miniature XAM-3D, R-tec instruments, San Jose, CA, USA). The nano-indentation tester by Anton Paar (Graz, Austria) was used to determine the nano-hardness and elastic modulus of the composite coatings. The CSM ball-on-disk friction tester was used to test the friction and wear behaviors of the coatings at room temperature. The steel balls were slid across the coatings under 10 N load at a speed of 10 cm/s. The amplitude was 2.5 mm, and the friction distance was 100 m. In this work, all the mechanical and tribological tests were performed at least five times for each POSS-PI-based composite coating.

## 3. Results

### 3.1. Characterization of the POSS-PI-Based Gradient Coatings

**Figure 2:**
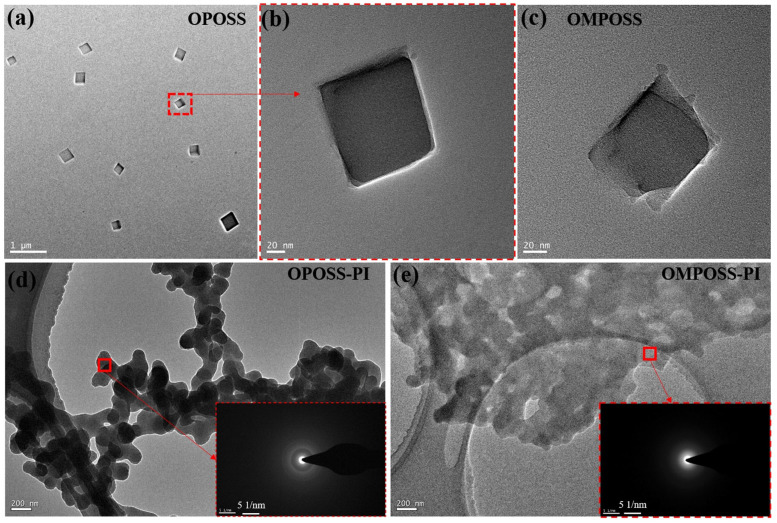
TEM images of POSS (**a**–**c**) and POSS-PAA-based composites (**d**,**e**). The insets show the SAED patterns of the corresponding samples.

**Figure 3:**
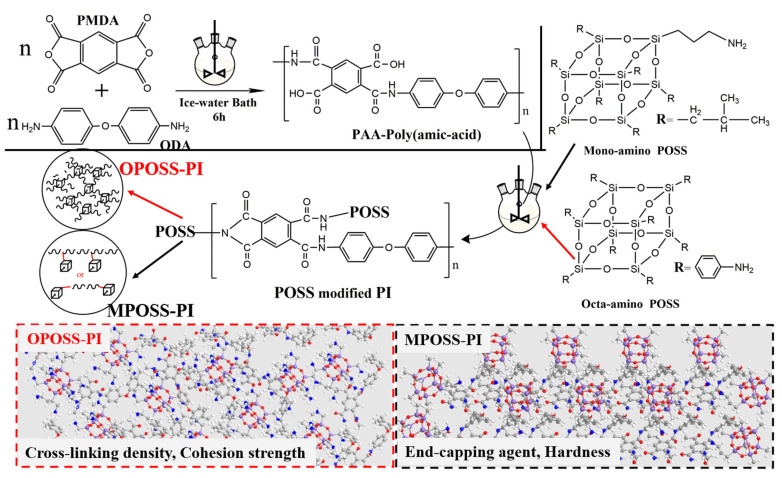
Schematic illustration of the preparation procedure for the POSS-PI composites.

Figure 2 illustrates typical transmission electron microscopy (TEM) photograph and selected area electron diffraction (SAED) patterns of the POSS and POSS-PI-based composites. It can be seen that the POSS shows a cube microstructure, with an average size of about 100 nm (Figure 2a–c). Unlike those of OMPOSS-PI composites, the morphologies of OPOSS-PI composites are well-regulated, and their SAED pattern shows a typical ring-like pattern due to the highly cross-linked and well-regulated structure (Figure 2d,e). Figure 3 shows the synthesis routes of the two kinds of PI-based nano-composites modified by octa- or mono-amino POSS, respectively. In the PI synthesis experiment, amino POSS was covalently grafted through amide reactions onto polyamide acids (PAAs) containing carboxyl groups. According to the characteristics of POSS, octa-amino groups in POSS react with carboxyl groups in PAA to act as a cross-linking agent, thus forming OPOSS-PI composite with a “star-shaped” microstructure; in contrast, the mono-amino POSS acts as an end-capping agent and is mainly distributed on the coating surface, thus forming the self-stratifying gradient structure.

**Figure 4:**
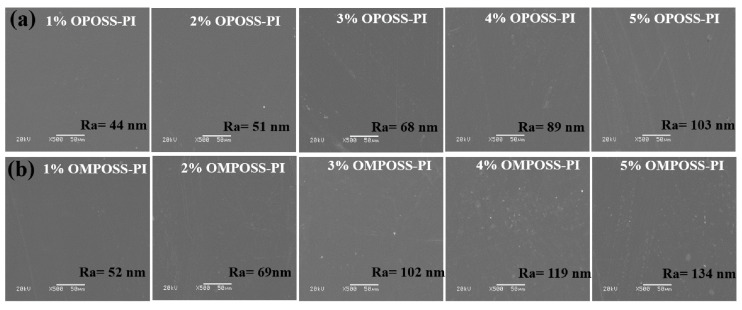
Surface SEM images and roughness of (**a**) OPOSS-PI and (**b**) OMPOSS-PI composites.

**Figure 5:**
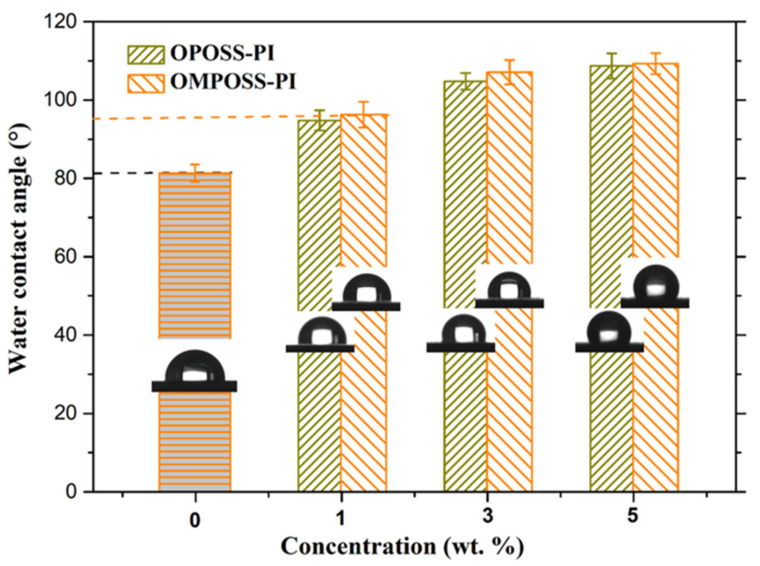
Water contact angle (°) of PI and OPOSS- and OMPOSS-modified composites.

The surface morphologies and roughness of the pure PI and POSS-modified composite coatings were investigated, as shown in Figure 4. It can be seen that a;4ll the composites showed smooth surfaces, and their roughness improved with increasing POSS content. For the OPOSS- or OMPOSS-modified PI-based composites, the increased roughness can be attributed to the surface migration of POSS nanomaterials [9]. Furthermore, the water contact angles of various PI-based composites were determined, and the results are shown in Figure 5. The results revealed that the pure PI composite exhibits a certain degree of wettability, and the angle improved with the increased POSS content corresponding to the increased surface roughness. In addition, the angle between OPOSS-PI and OMPOSS-PI shows slight difference. As a result, the surface migration of POSS on the coating surface increased the roughness and decreased the surface energy of the PI-based composite coatings, thereby improving the water contact angle.

**Figure 6:**
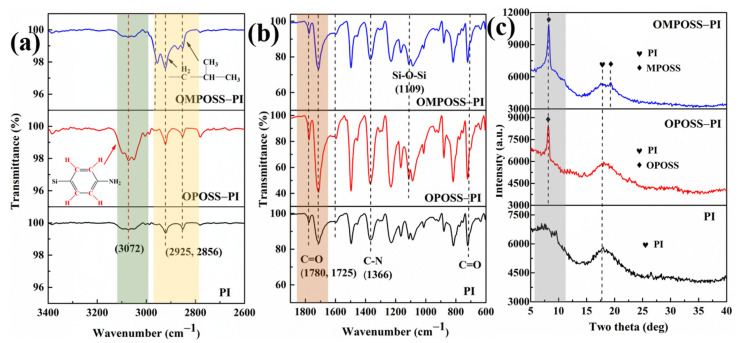
FTIR-ATR (**a**,**b**) and XRD (**c**) spectra of the PI, OPOSS-PI (5 wt. %), and OMPOSS-PI (5 wt. %) composites.

To study the bonding chemistry of POSS on the PI matrix, FTIR, XRD, and XPS measurements were conducted on the composites. As shown in Figure 6a,b, the infrared spectrum of the pure PI resin exhibits obvious C=O absorption peaks at 1780 and 1725 cm^−1^, and relatively weak absorption peaks associated with C-H bonds appeared at 3072, 2925, and 2856 cm^−1^. As expected, compared with that of pure PI (Figure 6b), the patterns of both the OPOSS-PI and OMPOSS-PI composites exhibit a Si-O-Si stretching peak at 1109 cm^−1^ [22]. Moreover, the FT-IR spectrum of octa-amino-phenyl POSS-PI reveals a clear phenyl stretching peak at 3072 cm^−1^, and in the infrared spectrum of the mono-amino hepta-isobutyl POSS-modified PI, an obvious C-H vibration peak appears at 2925 cm^−1^ [27]. In addition, the intensity of the C-N peaks of OPOSS-PI composites at 1366 cm^−1^ is slightly increased compared with those for PI and OMPOSS-PI. The XRD curves of the POSS-PI composites were also obtained, as shown in Figure 6c. For the PI resin, there is only one amorphous peak in the spectrum, while in the POSS-modified PI resin, obvious crystalline peaks appear at 9° and 15.6°, corresponding to OPOSS and OMPOSS. In addition, it can be seen from the spectra that the intensity of the crystalline peak for OMPOSS-PI is significantly higher than that for OPOSS-PI.

**Figure 7:**
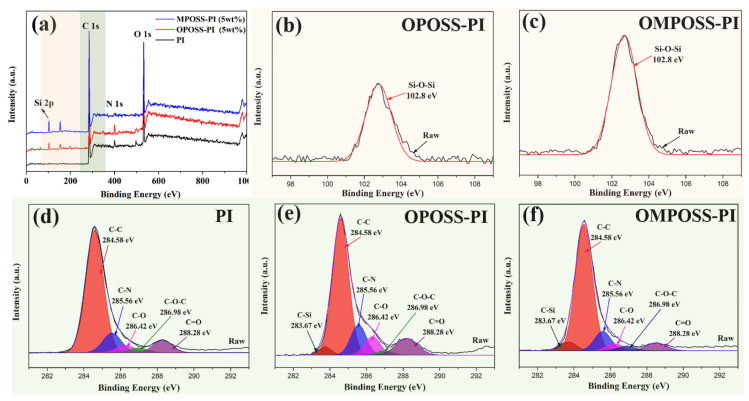
XPS survey spectra (**a**) and high-resolution Si 2p (**b**,**c**) and C 1s (**d**–**f**) XPS spectra of the PI, OPOSS-PI, and OMPOSS-PI composites.

The same result could be obtained via XPS analysis of the composites, as shown in Figure 7. Some peaks appeared in the survey XPS spectra for the PI composites, corresponding to C 1s, N 1s, and O 1s, respectively. However, the additional peaks at around 102.8 eV (Si 2p) and 152 eV (Si 2s) appeared for both the OPOSS-PI and OMPOSS-PI composites (Figure 7a). In addition, it is notable that the silicon content of OMPOSS-PI was significantly higher than that of OPOSS-PI (Figure 7b,c). The C 1s spectra were Gaussian curve-fitted to track the changes in the functional groups during the synthesis process. The peaks could be split into peaks centered at 283.67, 284.58, 285.56, 286.42, 286.98, and 288.28 eV, which correspond to C-C, C-N, C-O, C-O-C, and C=O bonds, respectively [28]. Unlike the spectrum for pure PI, those for the POSS-PI composites exhibited a peak associated with C-Si bonds (Figure 7d,e), and the results were in accordance with the FTIR spectra. Furthermore, the content of the C-N bonds increased greatly compared with OMPOSS-PI, as shown in Table 2, which can be attributed to the eight amino groups in OPOSS. The above results revealed that octa- and mono-amino POSS had been successfully grafted onto the PI matrix.

### 3.2. Mechanical Properties of OPOSS-PI and OMPOSS-PI Composites

**Figure 8:**
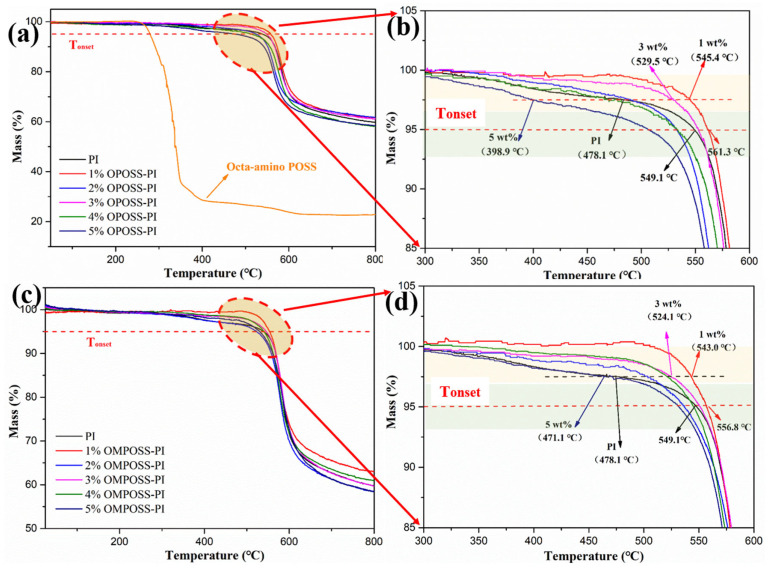
TGA curves of pristine PI, (**a**,**b**) OPOSS-PI, and (**c**,**d**) OMPOSS-PI composites.

The thermal stability of a polymer is also an important indicator of its engineering application potential. Figure 8a,c show the TGA curves for the OPOSS- and OMPOSS-modified PI materials from 25 to 800 °C in a N_2_ atmosphere. The corresponding temperature at which the mass loss reached 5% was defined as the onset degradation temperature (Tonset) of the system. As shown in Figure 8b,d, the heat resistance of the PI-based composites was significantly enhanced by the addition of POSS, and the Tonset increased from 549.1 °C to 561.3 and 556.8 °C, respectively. It is worth noting that, at a mass loss of 2.5%, the rangeability of degradation temperature became more pronounced. Figure 8b reveals that the introduction of octa-amino POSS improved the thermal stability of the resin. In particular, when the addition amount was 1 wt.%, the thermal stability of OMPOSS-PI increased by 67.3°, while the Tonset increased by 62.9° (Figure 8d). Such an expected enhancement can be mainly attributed to the microstructure and heat resistance performance of POSS [29]. The role of the cross-linking agent improves the cross-linking density and interfacial strength of the resin, and because the inorganic skeleton of the Si-O-Si bond in POSS hinders the thermal movement of the molecular chain, their joint action can effectively delay the production of volatile components in the PI resin.

**Figure 9:**
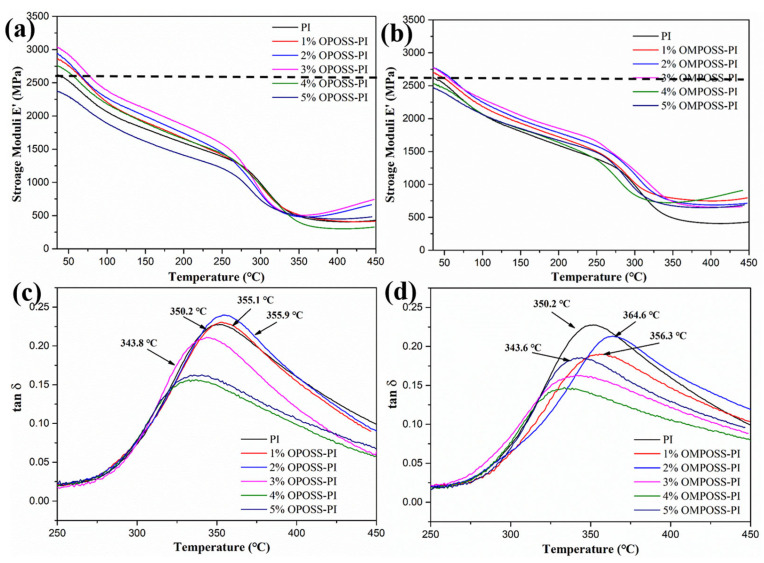
Storage modulus E’ and tan δ of (**a**,**c**) OPOSS-PI and (**b**,**d**) OMPOSS-PI.

The thermo-mechanical properties of the POSS-PI-based composites were also investigated by dynamic mechanical analysis (DMA) tester, and the results are presented in Figure 9. In Figure 9a, the storage modulus (E’) of the composites show a clear rising trend with increasing octa-amino POSS content, especially for the modified PI-based composites with 3 wt. % POSS. This result indicates that the addition of OPOSS greatly enhanced the cohesive energy of the PI-based coatings [28]. In Figure 9c, the loss tangent (tan δ) of OPOSS-PI shows the glass transition temperature (Tg) values. The Tg of OPOSS-PI composites rose and then fell with increasing OPOSS content. Obviously, the glass transition temperature of the 2 wt. % POSS-PI nano-composite was higher than that of the pure PI resin, which further confirmed that the addition of octa-amino POSS enhanced the thermal stability of the PI resin [29,30]. In addition, the DMA data for OMPOSS-PI was similar to that of OPOSS-PI composites (Figure 9b,d). From the perspective of its storage modulus E’ and glass transition temperature, the storage modulus and Tg values increased slightly when the content of OMPOSS was 2 wt. %.

**Figure 10:**
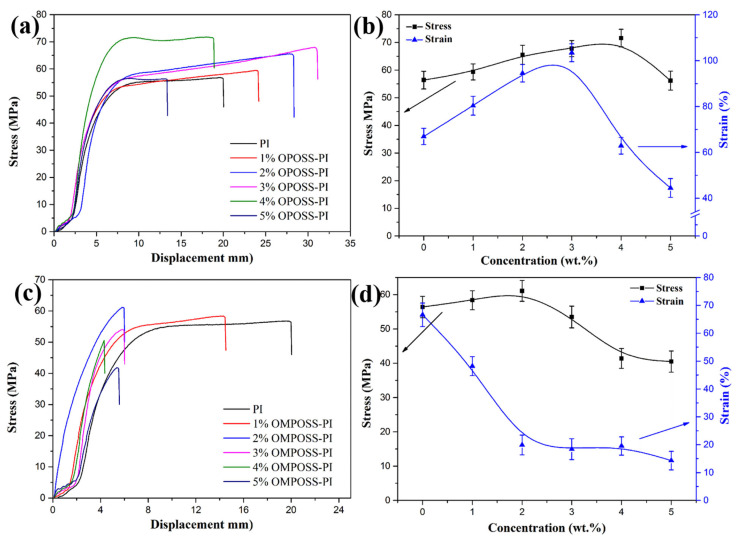
Typical load–displacement curves (**a**,**c**) and the tensile strength and elongation at break (**b**,**d**) of pure PI, OPOSS-PI (**a**,**b**), and OMPOSS-PI (**c**,**d**) composites.

The above analysis of dynamic thermo-mechanical properties shows that the introduced OPOSS acted as a cross-linking agent and effectively improved the cohesive energy of the PI-based composites, giving the composite better mechanical properties. Figure 10 depicts the tensile performance of the pure PI resin, OPOSS-PI, and OMPOSS-PI composites. As shown in Figure 10a, the tensile strength and elongation at break of the OPOSS-PI composites increased significantly as the content of OPOSS increased. In particular, the OPOSS-PI composites with 3 or 4 wt. % POSS content showed the highest tensile strength, which is 28.6% higher than that of pure PI. Moreover, the elongation at break of 3 wt. % OPOSS-PI was improved by 63.5% compared with that of pure PI (Figure 10b). As expected, the overall mechanical properties were superior to those of PI-based composites filled with other analogs, demonstrating the outstanding structural advantage of octa-amino POSS [31]. Additionally, as the content of OMPOSS increased, the tensile strength and elongation at break of OMPOSS-PI increased slightly and decreased significantly, respectively, as shown in Figure 10c,d. The change in tensile strength resulted from the improved cross-linking density and interfacial strength caused by OPOSS, while the decrease in elongation could be attributed to the end-capping effect of MPOSS.

**Figure 11:**
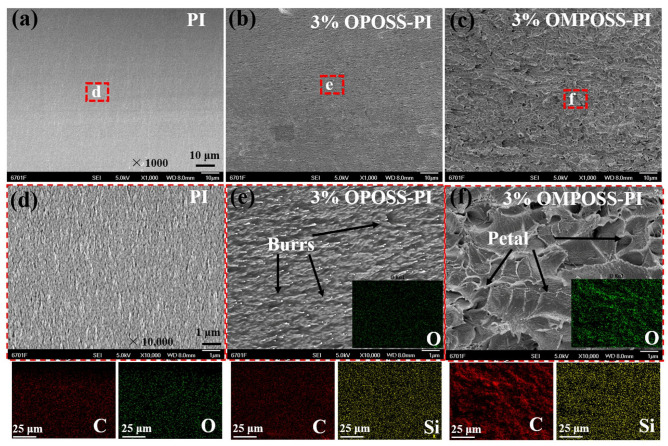
SEM morphologies of the fractured cross-sectional surfaces of pure PI (**a**,**d**), OPOSS-PI (**b**,**e**), and OMPOSS-PI (**c**,**f**) composite films.

Figure 11 shows the electron microscope morphologies (SEM) and EDS mapping of the stretched cross-sectional surfaces. The fracture surface of the pure PI resin was very flat, and no broken burrs appeared (Figure 11a,c). Meanwhile, on the fracture surface of the OPOSS-PI composite, there were many fracture burrs (Figure 11b,d), which can be attributed to the increased cross-linking density of OPOSS [28,32]. In addition, these burrs had a well-regulated and homogenous distribution, which indicates that OPOSS was well-dispersed in the PI matrix. However, the fracture surface of the OMPOSS-PI gradient composites showed a significantly different morphology (Figure 11c), with many irregular and different cracks that appeared on the surface, showing a “petal shape” (Figure 11f). This phenomenon may have resulted from the discontinuous chains and broken microstructures caused by unevenly distributed MPOSS in the PI matrix. In addition, the EDS mapping of carbon and silicon elements was consistent with the fractured surface morphologies.

**Figure 12:**
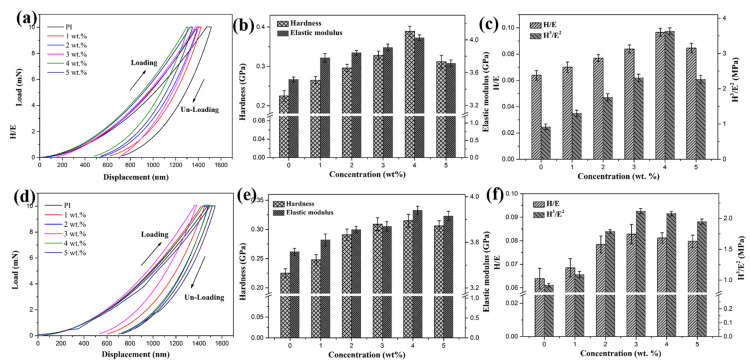
The load–displacement curves (**a**,**d**), H and E values (**b**,**e**), and H/E and H^3^/E^2^ values (**c**,**f**) of the (**a**–**c**) OPOSS-PI and (**d**–**f**) OMPOSS-PI hybrid composite coatings.

To study the effect of POSS content on the surface hardness of PI composites, nano-indentation tests were performed, and the results are shown in Figure 12. In the load–displacement curves for OPOSS-PI (Figure 12a), it is apparent that the maximum displacement (indentation depth) gradually decreased as the content of OPOSS increased. Figure 12b shows the nano-hardness and elastic modulus of the OPOSS-PI composites. Both the nano-hardness and elastic modulus of the composites increased with OPOSS content up to 4 wt.%, beyond which its hardness and elastic modulus decreased. The introduction of an appropriate amount of POSS increases the cross-linking density of the resin, thereby improving its mechanical properties, but the excess POSS causes a certain degree of self-polymerization. The previous research has indicated that the ratios H/E and H^3^/E^2^ are more appropriate than the single indentation hardness and elastic modulus for predicting the durability and plastic deformation resistance of a coating [33]. These two parameters, which are closely related to tribological behavior and resistance to mechanical failure, are very important for measuring the strength of the coating under local dynamic loads. In particular, a coating with higher H/E and H^3^/E^2^ values will have greater abrasion resistance and plastic deformation resistance. It can be seen from Figure 12c that as the content of OPOSS increased, the OPOSS-PI composites exhibited higher value of H/E and H^3^/E^2^. This proves that the abrasion and plastic deformation resistance of the OPOSS-PI composites was effectively improved, especially when the OPOSS content was 4 wt.%, which resulted in the best abrasion resistance. However, the maximum and residual displacements of OMPOSS-PI gradually decreased from 1515 ± 35 and 726 ± 25 nm to 1310 ± 30 and 537 ± 15 nm (Figure 12d). Moreover, the indentation hardness, elastic modulus, H/E and H^3^/E^2^ ratios also showed a similar tendency compared to those of the OPOSS-PI composites (Figure 12e,f), and the 4 wt. % OMPOSS-PI composites exhibited the highest value. Notably, the indentation resistance, hardness, and H/E values for the OMPOSS-PI gradient composites were all higher than those for the OPOSS-PI composites. Thus, the enhanced resistance to plastic deformation and wear resistance of these composites is attributed to the improved hardness caused by the gradient structures [34].

**Figure 13:**
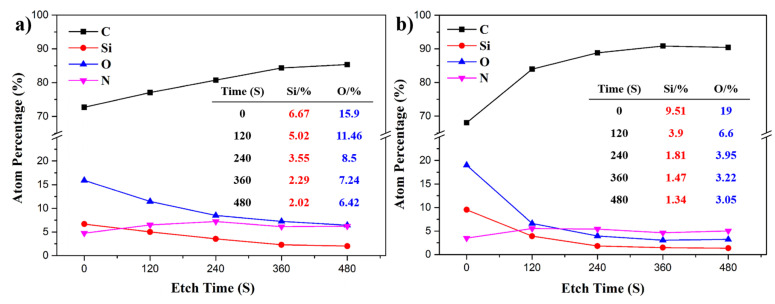
The relationship curves of atomic number percentage with etch time for the (**a**) 4 wt. % OPOSS-PI and (**b**) 4 wt. % OMPOSS-PI composites.

To investigate the different tendencies regarding the nano-hardness and elastic modulus of the POSS-PI composites, the surfaces of the POSS-PI self-stratifying gradient composites were etched with an Ar+ ion beam in a continuous period, and then the element composition at five different stages were determined by means of XPS. Figure 13 depicts a line graph of change in the atomic number percentage with the prolonged etch times. It can be seen that for both OPOSS and OMPOSS-modified PI composites, the atomic percentage of C element first increased with the prolonged etch time, while the atomic percentages of Si and O elements decreased, as the etch time increased. It is worth noting that the atomic number percentages of Si (9.51%) and O (19%) on the surface of OMPOSS-PI were higher than those for OPOSS-PI (6.67% and 15.9%). After 480 s, the atomic number percentage of Si of OPOSS-PI decreased gradually to 2.02%, but that of OMPOSS-PI decreased quickly to 1.34%. These results can be attributed to the surface migration effect and structure character of POSS. The octa-amino POSS was introduced into PI to act as a cross-linking agent and was distributed evenly in the PI matrix. In contrast, the mono-phenyl POSS was introduced into PI acting as an end-capping agent, which resulted in its surface accumulation [24,35].

### 3.3. Tribological Properties of POSS-PI Gradient Composite Coatings

**Figure 14:**
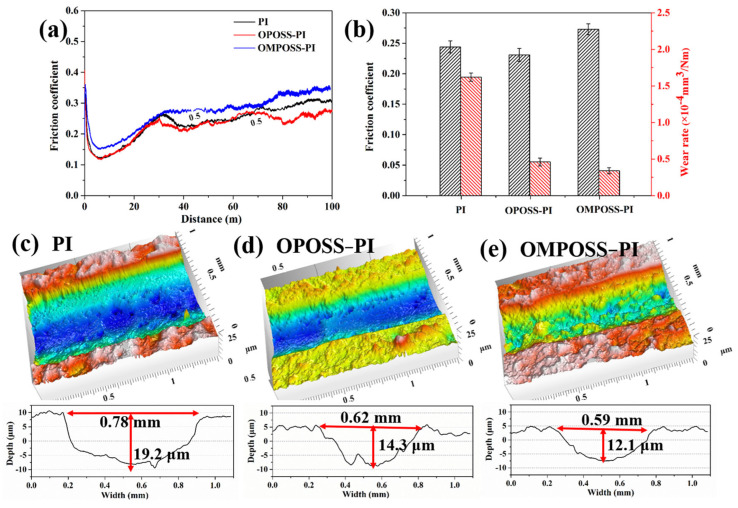
The coefficient of friction curves (**a**), wear rates (**b**), and corresponding 3D morphologies and cross-sectional curves of the wear tracks (**c**–**e**) of the pure PI, 4 wt. % OPOSS-PI, and 4 wt. % OMPOSS-PI composites.

Apart from the thermal stability and mechanical properties of the POSS-PI composites, we also studied the influence of its structures on the friction and wear behaviors of the POSS-PI-based gradient composite coatings. Figure 14a shows the friction coefficient curves of the corresponding composite coatings. The friction coefficient of OPOSS-PI was the smallest among the three coatings. The corresponding wear rates were also determined, and the results are shown in Figure 14b. The PI coating suffered severe wear (1.95 × 10^−4^ mm^3^·N^−1^·m^−1^), but the wear rate of the OMPOSS-PI gradient coating decreased significantly (0.41 × 10^−4^ mm^3^·N^−1^·m^−1^) compared with that of the PI coating. The dramatic decrease further demonstrates the enhanced wear resistance of the PI-based composite coatings. Figure 14c–e show the three-dimensional (3D) morphologies and corresponding cross-sectional profiles of the wear tracks of the PI-based composite coatings. As shown in Figure 14a, the wear track of the pure PI resin was the widest and deepest (width 0.78 mm and depth 19.2 μm). The widths and depths of the wear tracks on the OPOSS-PI (0.62 mm and 14.3 μm) and OMPOSS-PI (0.59 mm and 12.1 μm) composite coatings were greatly decreased compared with those of pure PI (Figure 14d,e), indicating that the gradient structure of OMPOSS-PI significantly improved the wear resistance of the PI coating.

**Figure 15:**
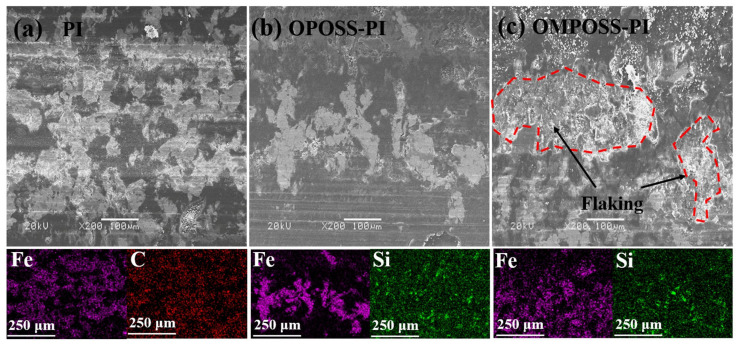
SEM morphologies and EDS analyses of the worn surfaces of the (**a**) pure PI, (**b**) 4 wt. % OPOSS-PI, and (**c**) 4 wt. % OMPOSS-PI composite coatings.

Figure 15 shows the SEM morphologies of the worn surfaces of pure PI, OPOSS-PI, and OMPOSS-PI coatings and the corresponding EDS element analysis results. It can be seen that the Fe element was distributed across almost the entire worn surface of the pure PI coating (Figure 15a), which revealed that the coating suffered severe wear. Compared with the pure PI coating, the wear degree of the OPOSS-PI coating was greatly reduced, and the surface of the wear scar was smoother (Figure 15b). Additionally, the worn surface of OMPOSS-PI coatings is obviously different. The composite coating showed obvious laminar peeling inside the worn surface (Figure 15c). The corresponding Fe elements were distributed on the surface, and there was obvious accumulation in the Si element distribution. This could be attributed to the fact that the end-capping effect resulted in the uneven distribution of MPOSS and a low interface adhesion strength inside the coating. However, the peeled-off POSS-PI will transfer to the surface of the counterpart ball to form a lubricating film, thereby reducing the further wear of the coating. As a result, the evenly distributed Si-O-Si inorganic framework, as shown in the Si element mapping, endowed PI-based composites with great load-bearing capacity, and the results described above reveal that the addition of OMPOSS effectively improved the tribological properties.

## 4. Conclusions

In this work, both octa- and mono-amino POSS (OMPOSS) with a unique cage-like microstructure were chemically bonded onto the PI matrix, and the OMPOSS-PI-based self-stratifying gradient composite coatings were thereby successfully fabricated. The Si elements were distributed gradually from the coating surface to the interior. Compared with those of pure PI, the introduction of OMPOSS significantly improved the thermal stability and mechanical properties of the PI-based composites, which can be attributed to the high cross-linking density resulting from the multi-functional amino groups. Notably, the tensile strength and nano-hardness apparently improved by 28.6% and 68.4%. Additionally, the OMPOSS-PI self-stratifying gradient coatings also exhibit excellent wear resistance, with a low wear rate at 0.41 × 10^−4^ mm^3^·N^−1^·m^−1^, which decreased by 78.9% greatly compared with pure PI coating. This dense structure and hollow cage-like inorganic framework consists of Si-O-Si bonds, which provide the PI-based coating with excellent load-bearing capacity, thereby improving these tribological properties.

## Figures and Tables

**Table 1:** Weight contents of the POSS-PI-based gradient composite coatings.

Coatings	Materials (g)				Content (wt. %)
	PMDA	ODA	Mono-POSS	Octa-POSS	
PI	1.65	1.5	0	0	0
OPOSS-PI	1.65	1.5	0	0.03	1
				0.06	2
				0.09	3
				0.12	4
				0.15	5
OMPOSS-PI	1.65	1.5	0.015	0.015	1
			0.03	0.03	2
			0.045	0.045	3
			0.06	0.06	4
			0.075	0.075	5

**Table 2:** XPS data of the C 1s bond contents (%) of the composite coatings.

Coatings	Content (%)					
	C-Si	C-C	C-N	C-O	C-O-C	C=O
PI	0	73.32	11.75	4.38	2.76	7.79
OPOSS-PI	3.08	62.95	13.34	8.17	2.01	10.45
OMPOSS-PI	5.03	72.51	10.62	3.92	5.31	2.61

## Data Availability

The original contributions presented in this study are included in the article. Further inquiries can be directed to the corresponding authors.
